# Supplementary material for: Dermatologist-like explainable AI enhances melanoma diagnosis accuracy: eye-tracking study
Source: Nat Commun. 2025 May 21;16:4739. doi: 10.1038/s41467-025-59532-5 (PMC12095463; doi:10.1038/s41467-025-59532-5)
Supplement: Supplementary file 1 — Supplementary Information [file 41467_2025_59532_MOESM1_ESM.pdf]

Supplementary Information

Dermatologist-like explainable AI enhances melanoma diagnosis accuracy: eye-tracking study

a

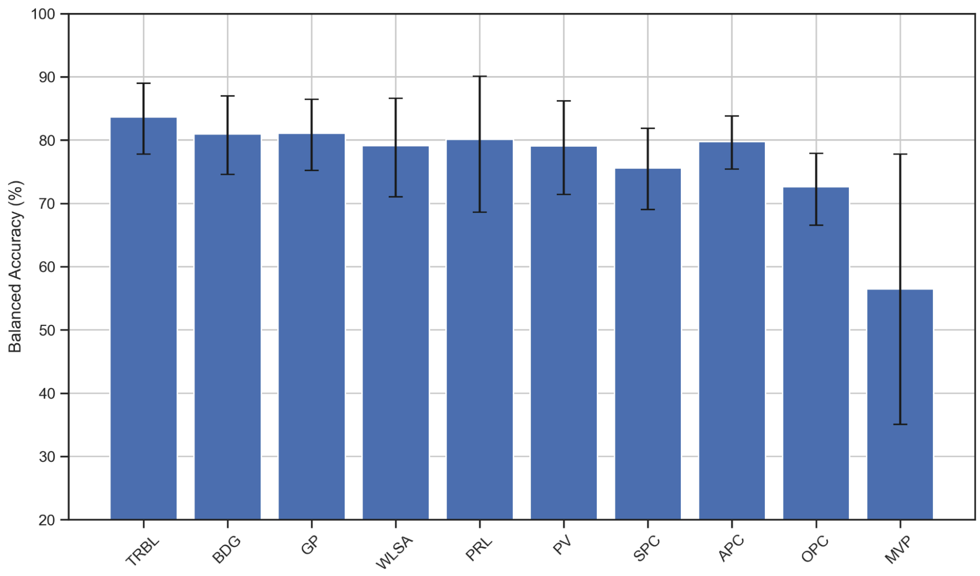

Supplementary Fig 1: Balanced accuracies of the characteristics.

a, TRBL: Thick reticular or branched lines. BDG: Black dots or globules. GP: Gray patterns. WLSA: White lines or white structureless area. PRL: Pseudopods or radial lines at the lesion margin. PV: Polymorphous vessels. SPC: Symmetrical combination of patterns and/or colors. OPC: Only one pattern and/or color. MVP: Monomorphic vascular patterns. Data are presented as mean values and the error bars represent bootstrapped confidence intervals derived from 1000 samples. n=216 images.

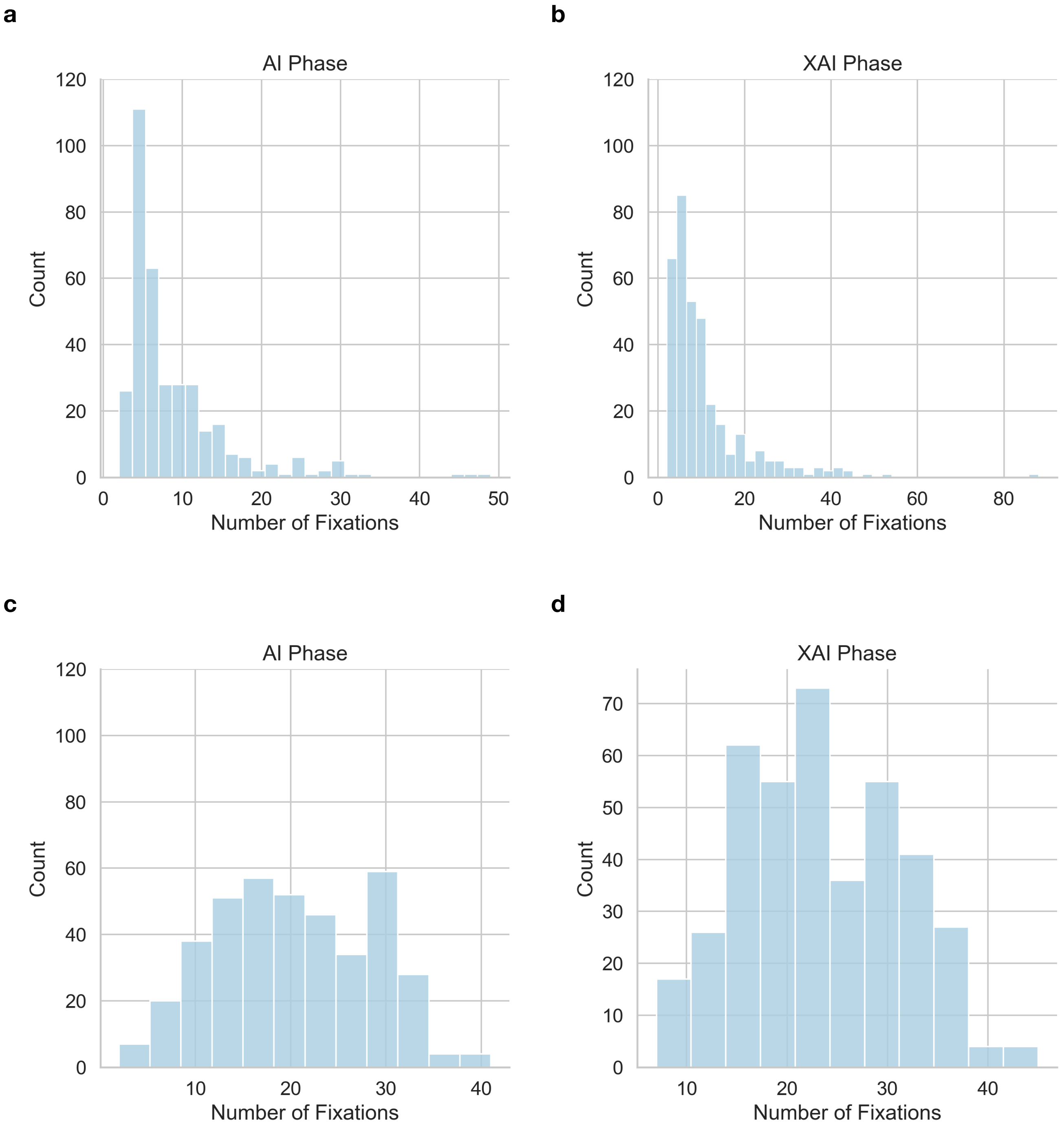

Supplementary Fig 2: Distributions of fixation data.

a, b: Distribution of artificial intelligence (AI) and explainable artificial intelligence (XAI) phase fixation counts in the web-based portion of the study (n=353). **c, d:** Distribution of AI and XAI phase fixation counts in the device-based portion of the study (n=400). Data are presented as counts. Data are presented as counts per bin.

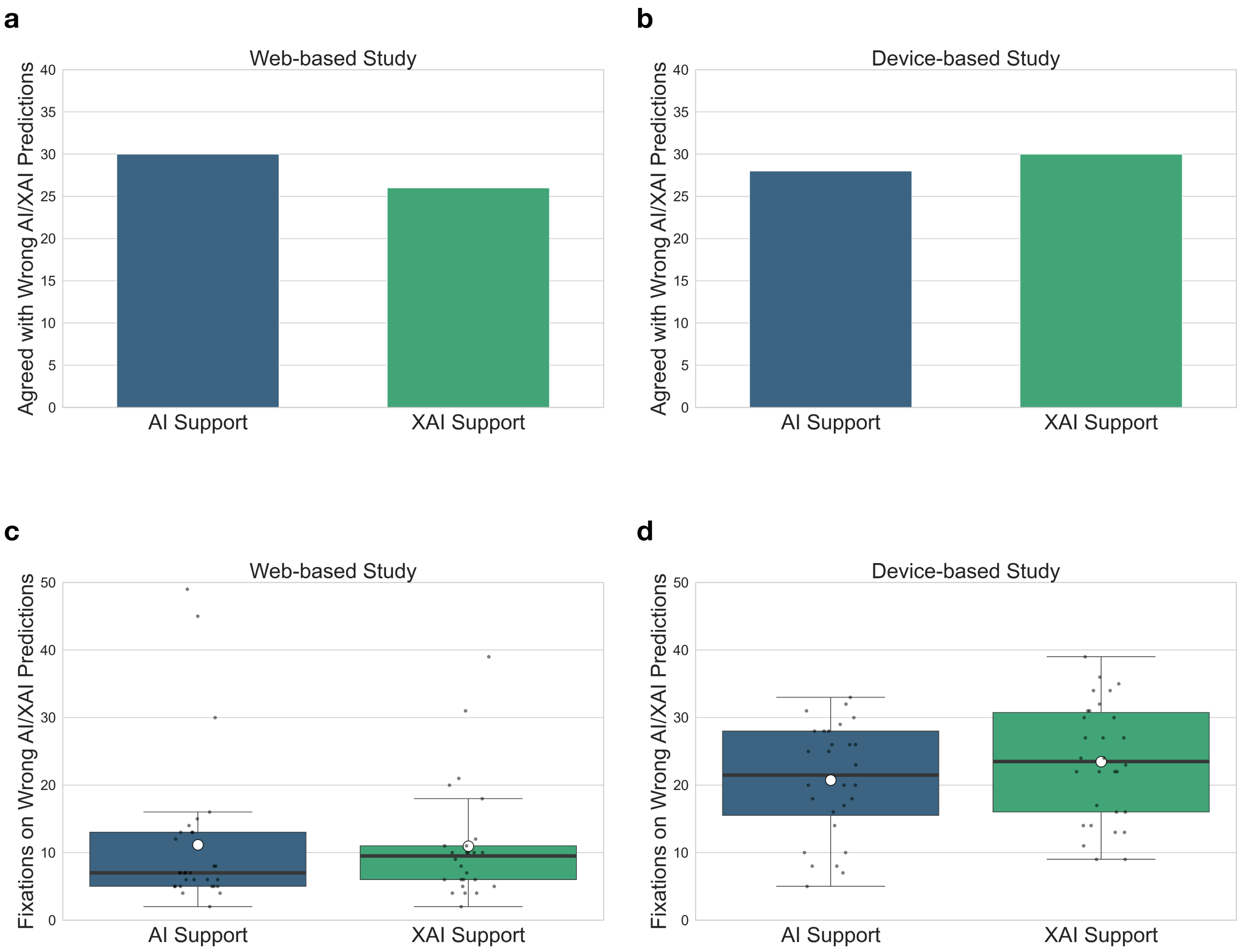

Supplementary Fig 3: Wrong AI/XAI Predictions.

a, b: Counts of dermatologist agreement with incorrect artificial intelligence (AI)/ explainable artificial intelligence (XAI) predictions in the web-based study (n=56) (**a**) and the device-based study (n=58) (**b**). No significant difference was observed between the AI Support and XAI Support phases in either study. Data are presented as the number of times incorrect predictions were agreed with. **c, d:** Comparison of fixation counts on incorrect AI/XAI predictions in the web-based study (n=56) (**c**) and the device-based study (n=58) (**d**). No significant difference was observed between the AI Support and XAI Support phases in either study (Mann-Whitney U Test; web-based: P=0.69, device-based: P=0.69). The horizontal line on each box denotes the median value and the white dot denotes the mean. The upper and lower box limits denote the 1st and 3rd quartiles, respectively, and the whiskers extend from the box to 1.5 times the interquartile range.

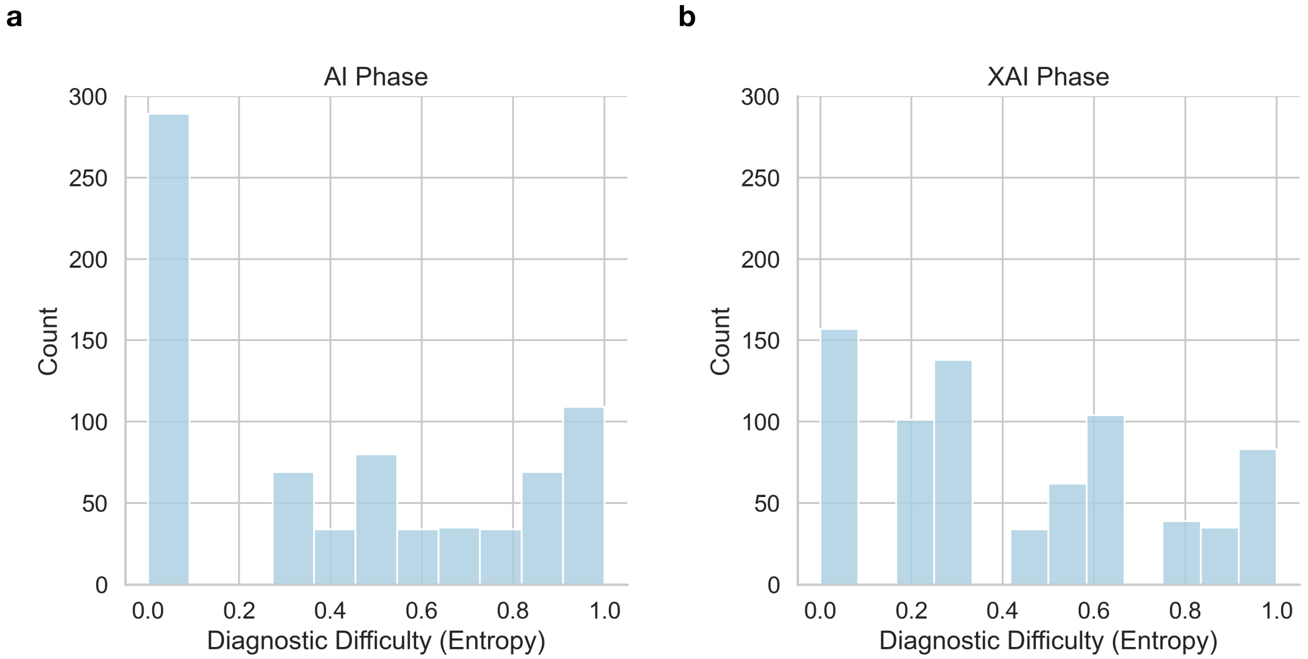

Supplementary Fig 4: Distributions of diagnostic difficulty.

a, b: Distribution of diagnostic difficulty of the images used in the artificial intelligence (AI) (n=753) and explainable artificial intelligence (XAI) (n=753) phases, computed by the entropy of the predictions. Higher entropy indicates greater disagreement among the dermatologists on the diagnosis of an image. Data are presented as counts per bin.

Experience in Years	Mean Balanced Accuracy (AI Phase)	Mean Balanced Accuracy (XAI Phase)	Median Balanced Accuracy (AI Phase)	Median Balanced Accuracy (XAI Phase)	Num Dermatologists
1 - 3 Years	81.8	83.9	81.3	87.5	12
3 - 5 Years	81.3	83.9	81.3	87.5	14
5 - 10 Years	77.0	79.3	81.3	81.3	13
> 10 Years	80.0	80.4	81.3	81.3	22

Supplementary Table 1: Dermatologists balanced accuracies and experience levels

AI = artificial intelligence; XAI = explainable artificial intelligence. n=61

Subset	Two-sided Spearman Correlation	P-value
Full	-0.42	0.003 (46 dermatologists)
Web-based	-0.07	0.74 (23 dermatologists)
Device-based	-0.78	<0.001 (23 dermatologists)

Supplementary Table 2: Correlations of fixations with dermatologists experience levels.

n=46

Subset	Two-sided Spearman Correlation	P-value
Full	-0.42	0.003 (46 dermatologists)
Web-based	-0.07	0.74 (23 dermatologists)
Device-based	-0.78	<0.001 (23 dermatologists)

Supplementary Table 3: Fixation correlations with dermatologists' experience levels without adjusting for outlier fixations.

n=46

	Discarded Participants
**Phase 1**	4
**Phase 2**	1

Supplementary Table 4: Participants eye tracking reliability distribution.

Number of discarded participants in each phase due to unreliable eye tracking data. Reliability was estimated based on webcam sampling rate and amount of missing gaze data (participant not looking at their screen).

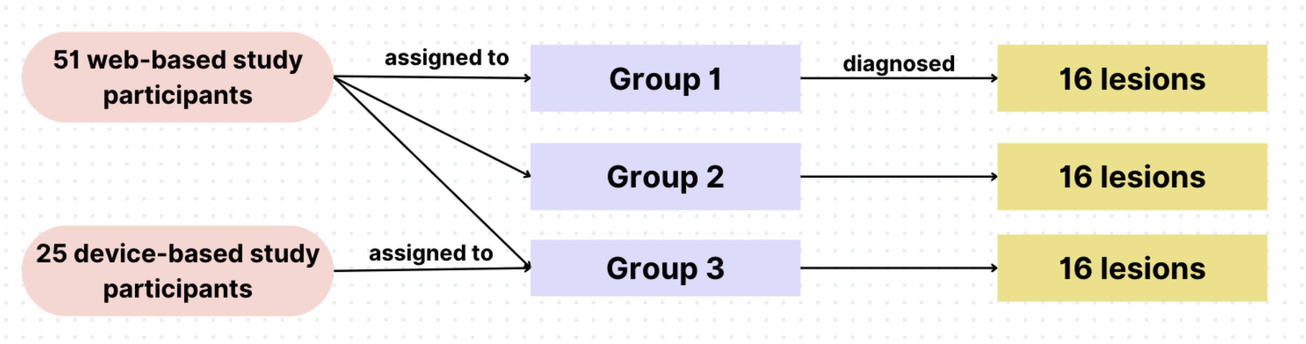

Supplementary Fig 5: Participant flow.
