# Supplementary material for: Dermatologist-like explainable AI enhances melanoma diagnosis accuracy: eye-tracking study
Source: Nat Commun. 2025 May 21;16:4739. doi: 10.1038/s41467-025-59532-5 (PMC12095463; doi:10.1038/s41467-025-59532-5)
Supplement: Supplementary file 2 — Reporting Summary [file 41467_2025_59532_MOESM2_ESM.pdf]

nature portfolio

Corresponding author(s): Titus J. Brinker

Last updated by author(s): Mar 12, 2025

# Reporting Summary

Nature Portfolio wishes to improve the reproducibility of the work that we publish. This form provides structure for consistency and transparency in reporting. For further information on Nature Portfolio policies, see our Editorial Policies and the Editorial Policy Checklist.

## Statistics

For all statistical analyses, confirm that the following items are present in the figure legend, table legend, main text, or Methods section.

| n/a | Confirmed | |
|---|---|---|
| ☐ | ☒ | The exact sample size ($n$) for each experimental group/condition, given as a discrete number and unit of measurement |
| ☐ | ☒ | A statement on whether measurements were taken from distinct samples or whether the same sample was measured repeatedly |
| ☐ | ☒ | The statistical test(s) used AND whether they are one- or two-sided<br>*Only common tests should be described solely by name; describe more complex techniques in the Methods section.* |
| ☐ | ☒ | A description of all covariates tested |
| ☐ | ☒ | A description of any assumptions or corrections, such as tests of normality and adjustment for multiple comparisons |
| ☐ | ☒ | A full description of the statistical parameters including central tendency (e.g. means) or other basic estimates (e.g. regression coefficient) AND variation (e.g. standard deviation) or associated estimates of uncertainty (e.g. confidence intervals) |
| ☐ | ☒ | For null hypothesis testing, the test statistic (e.g. $F$, $t$, $r$) with confidence intervals, effect sizes, degrees of freedom and $P$ value noted<br>*Give P values as exact values whenever suitable.* |
| ☒ | ☐ | For Bayesian analysis, information on the choice of priors and Markov chain Monte Carlo settings |
| ☒ | ☐ | For hierarchical and complex designs, identification of the appropriate level for tests and full reporting of outcomes |
| ☐ | ☒ | Estimates of effect sizes (e.g. Cohen's $d$, Pearson's $r$), indicating how they were calculated |

*Our web collection on statistics for biologists contains articles on many of the points above.*

## Software and code

Policy information about availability of computer code

| | |
|---|---|
| Data collection | The web-based survey tool Pabbly (https://forms.pabbly.com), the web-based eye tracking software RealEye (https://www.realeye.io), and the dedicated eye tracking device Pupil Labs Core (https://pupil-labs.com/products/core) were used to collect data. |
| Data analysis | All code was written in Python (3.9.9). PyTorch (1.10.0), PyTorch Lightning (1.5.10), Albumentations (1.0.3), NumPy (1.22.2), Pandas (1.4.0), SciPy (1.8.0), OpenCV (4.5.5), Scikit-learn (1.1.0), Matplotlib (3.1.1), and Seaborn (0.11.2) were used for image processing, model development and training, data analysis, and visualization. |

For manuscripts utilizing custom algorithms or software that are central to the research but not yet described in published literature, software must be made available to editors and reviewers. We strongly encourage code deposition in a community repository (e.g. GitHub). See the Nature Portfolio guidelines for submitting code & software for further information.

## Data

Policy information about availability of data

All manuscripts must include a data availability statement. This statement should provide the following information, where applicable:

- Accession codes, unique identifiers, or web links for publicly available datasets
- A description of any restrictions on data availability
- For clinical datasets or third party data, please ensure that the statement adheres to our policy

The data generated in our study, which includes the pseudonymized reader study data and the fixations data are accessible on Figshare: https://figshare.com/

s/5f0b0f18c20f0a850dc7. The HAM10000 dataset is publicly available and can be accessed here: https://dataverse.harvard.edu/dataset.xhtml?persistentId=doi:10.7910/DVN/DBW86T. External research projects may request access to the prospectively-collection additional dataset used in our study, specifically for the purpose of advancing skin (cancer) research. Access is granted following an application and approval process managed by the SCP Data Protection Committee, which evaluates requests based on criteria such as alignment with patient consent, a valid ethics vote, and other relevant requirements (i.e., non-commercial (skin) cancer research). All remaining data is publicly available. Commercial use of the data is prohibited.

## Research involving human participants, their data, or biological material

Policy information about studies with human participants or human data. See also policy information about sex, gender (identity/presentation), and sexual orientation and race, ethnicity and racism.

| | |
|---|---|
| Reporting on sex and gender | We did not collect any data on sex and gender of the clinicians participating in our reader study. The focus of the study was to investigate how dermatologists interact with explainable AI. We assume that gender does not play a role in this diagnostic behavior. Therefore, the exclusion of the gender aspect was a deliberate choice as we aimed to collect as little identifying personal data as possible. |
| Reporting on race, ethnicity, or other socially relevant groupings | We did not collect any data on race, ethnicity, or any other socially relevant groups of the clinicians participating in our reader study. |
| Population characteristics | The participants were had diverse levels of experience ranging from assistant dermatologists to clinic directors. |
| Recruitment | The participants were contacted via Email through our collaboration network and by using public contact data from the International Society for Dermoscopy website and from university clinic webpages. We also contacted participants from private clinics. As compensation, we offered them the option to be listed as a PubMed indexed collaborator on our work. Due to this line of recruitment, the reader study population is biased towards clinicians practicing in university clinics and interested in taking part in scientific research. The group of clinicians who practice dermoscopy in public hospitals and private practices is under-represented as they had less incentive to take part in the study. |
| Ethics oversight | Ethics approval was obtained from the ethics committee at the Technical University of Dresden (BO-EK-53012021), the Friedrich-Alexander University Erlangen-Nuremberg (69_21 Bc), the LMU Munich (21-0182), the University of Regensburg (20-2190-103), the Julius-Maximilians University Wuerzburg (293/20_z) and from the University Hospitals Mannheim (2020-656N) and Essen (20-9784-BO). Informed consent was collected from all participants. We did not collect any data on sex and gender of the clinicians participating in our reader study. As compensation, we offered them the opportunity to be credited as a collaborator of our work. |

Note that full information on the approval of the study protocol must also be provided in the manuscript.

# Field-specific reporting

Please select the one below that is the best fit for your research. If you are not sure, read the appropriate sections before making your selection.

☒ Life sciences ☐ Behavioural & social sciences ☐ Ecological, evolutionary & environmental sciences

For a reference copy of the document with all sections, see nature.com/documents/nr-reporting-summary-flat.pdf

# Life sciences study design

All studies must disclose on these points even when the disclosure is negative.

| | |
|---|---|
| Sample size | 76 dermatologists participated in the reader study. In accordance with the central limit theorem, we required a minimum sample size of 30 participants; the more clinicians that participated, the better. |
| Data exclusions | We excluded participants that dropped out during the study. |
| Replication | Replication was not performed. |
| Randomization | All participants were assigned to one of three groups. The German speaking participants were assigned to group 3 and the rest were randomly assigned to one of group 1 and 2. |
| Blinding | The participating clinicians were not aware of the existence of different groups, the group they were assigned to, or the distribution of melanoma and nevus images. |

# Reporting for specific materials, systems and methods

We require information from authors about some types of materials, experimental systems and methods used in many studies. Here, indicate whether each material, system or method listed is relevant to your study. If you are not sure if a list item applies to your research, read the appropriate section before selecting a response.

## Materials & experimental systems

| n/a | Involved in the study |
|---|---|
| ☒ | ☐ Antibodies |
| ☒ | ☐ Eukaryotic cell lines |
| ☒ | ☐ Palaeontology and archaeology |
| ☒ | ☐ Animals and other organisms |
| ☒ | ☐ Clinical data |
| ☒ | ☐ Dual use research of concern |
| ☒ | ☐ Plants |

## Methods

| n/a | Involved in the study |
|---|---|
| ☒ | ☐ ChIP-seq |
| ☒ | ☐ Flow cytometry |
| ☒ | ☐ MRI-based neuroimaging |

# Plants

Seed stocks

*Report on the source of all seed stocks or other plant material used. If applicable, state the seed stock centre and catalogue number. If plant specimens were collected from the field, describe the collection location, date and sampling procedures.*

Novel plant genotypes

*Describe the methods by which all novel plant genotypes were produced. This includes those generated by transgenic approaches, gene editing, chemical/radiation-based mutagenesis and hybridization. For transgenic lines, describe the transformation method, the number of independent lines analyzed and the generation upon which experiments were performed. For gene-edited lines, describe the editor used, the endogenous sequence targeted for editing, the targeting guide RNA sequence (if applicable) and how the editor was applied.*

Authentication

*Describe any authentication procedures for each seed stock used or novel genotype generated. Describe any experiments used to assess the effect of a mutation and, where applicable, how potential secondary effects (e.g. second site T-DNA insertions, mosiacism, off-target gene editing) were examined.*
